# Dentin Hypersensitivity and Gingival Recession: Impact of Root Surface Coverage—A Retrospective Study

**DOI:** 10.1111/jerd.70065

**Published:** 2025-12-03

**Authors:** Romain Ohanessian, Angéline Antezack, Alexandre Feuillette, Cyril Ferrier, Virginie Monnet‐Corti

**Affiliations:** ^1^ Faculté des Sciences Médicales et Paramédicales, École de Médecine Dentaire Aix‐Marseille Université Marseille France; ^2^ Assistance Publique‐Hôpitaux de Marseille (AP‐HM) Hôpital Timone, Service de Parodontologie Marseille France; ^3^ UMR D‐258 Microbes Evolution Phylogénie et Infection (MEPHI), Institut de Recherche Pour Le Développement (IRD), Aix‐Marseille Université, Provence‐Alpes‐Côte D'azur Marseille France

**Keywords:** clinical study, dentin hypersensitivity, dentin sensitivity, gingival recession, tooth root

## Abstract

**Objective:**

To assess whether root surface coverage after periodontal plastic surgery is associated with significant dentin hypersensitivity suppression.

**Materials and Methods:**

Included patients presented a significant dentin hypersensitivity (Schiff score ≥ 2) and gingival recession. Treatment consisted of periodontal plastic surgery for root coverage. At 6 months, significant dentin hypersensitivity prevalence was assessed. Surgical outcomes were evaluated by measuring the percentage of root surface coverage and the height of root coverage, both in pixels and millimeters, in two groups: teeth without significant dentin hypersensitivity and those with persistent significant dentin hypersensitivity.

**Results:**

Significant dentin hypersensitivity prevalence was 6.8% (95% CI [1.0%–12.5%]), with suppression in 93.2% of treated teeth 6 months postoperatively. Complete root surface coverage (CRSCpix) was achieved in 69.6% without dentin hypersensitivity, significantly higher than 0.0% in teeth with dentin hypersensitivity (*p* = 0.0041). Complete root height coverage in pix (CRHCpix) was 63.8% without dentin hypersensitivity versus (vs) 20.0% with DH (*p* = 0.0737), while complete root height coverage in mm (CRHCmm) was 76.8% without DH vs. 40.0% with dentin hypersensitivity (*p* = 0.1033). Mean root surface coverage in pixels (RSCpix) was 88.3% ± 19.7% without DH, significantly higher than 62.6% ± 28.2% with dentin hypersensitivity (*p* = 0.0031). Mean root height coverage in pixels (RHCpix) was 83.2% ± 28.3% without DH versus 68.8% ± 35.1% with dentin hypersensitivity (*p* = 0.0573), while mean root height coverage in millimeters (RHCmm) was 88.2% ± 24.7% without dentin hypersensitivity versus 73.3% ± 30.8% with dentin hypersensitivity (*p* = 0.0503). No significant differences were found for height‐based root coverage in pixels (RHCpix) and millimeters (RHCmm).

**Conclusion:**

Success of surgical root coverage, particularly the amount of root surface covered, could be a key factor in dentin hypersensitivity suppression.

## 1 Introduction

Dentin hypersensitivity (DH) has been defined as an acute, brief, or transient pain associated with dentin exposure, triggered by tactile, thermal, chemical, or osmotic stimuli, without being linked to other dental pathologies [1]. The assessment of DH relies on both objective measures, including cold air blast tests and tactile stimuli tests [2], and subjective measures like patient‐reported outcome measures (PROMs), such as the visual analog scale, verbal descriptors, and dichotomous reporting. DH predominantly affects adults [3]. For patients, bothersome or intolerably marked DH, prompting them to seek treatment, can often be identified by a Schiff test score of 2 or 3 [4]. Recently, West et al. estimated a prevalence of 40.6% among individuals and found that 29.0% of them had a Schiff score of 2 or 3 [5]. According to Goh et al., DH affects Oral Health‐related Quality of Life (OHRQoL) in patients undergoing supportive periodontal care with the extent of impact being associated with the severity of DH [6]. Systematic reviews have confirmed the negative effect of DH on OHRQoL, which can be improved with treatment [7, 8]. Furthermore, various treatments have been proposed, including occlusion of dentinal tubules [4, 9] and root coverage (RC) through periodontal plastic surgery.

In addition, photobiomodulation (PBM), using red light such as 660 nm, has shown significant efficacy for reducing DH [10].

Gingival recessions (GR) are considered a major contributing factor of DH, as they expose the cervical and root dentin [11, 12]. A recent European study reports a DH prevalence of 75.9% of participants, often associated with dental wear (97.6%) and the major risk factor GR (87.9%) [5]. Predisposing factors include a thin periodontal biotype, absence of attached gingiva, and reduced alveolar bone thickness [13]. Additional contributors, such as improper toothbrushing, oral piercings, orthodontic treatment, intrasulcular restorative margins, and chronic gingival inflammation have been widely studied [14, 15, 16]. In addition to DH, GR can lead to esthetic concerns, plaque accumulation, and an increased risk of root caries. The negative effects of GR on OHRQoL are largely mediated by dentin exposure and subsequent DH, which has been shown to decrease following periodontal plastic surgery, leading to improvements in OHRQoL [6].

Recent meta‐analysis showed that DH suppression was achieved in 70.8% of cases following RC surgery, confirming the effectiveness of this surgical approach [17]. Most of the reviewed studies have evaluated outcomes in terms of recession height in millimeters or the average percentage of root coverage based on clinical measurements. But measuring only height of recession does not accurately reflect the total root surface area exposed to painful stimuli [18]. The analysis of the exposed recession surface area (RSA), measured on digital photographs using the open‐source software ImageJ [19], provides a more comprehensive method for analyzing the amount of RC.

The aim of this study was to evaluate whether the extent of root surface coverage after periodontal plastic surgery influences the suppression of significant DH.

## 2 Materials and Methods

### 2.1 Patient Population

The retrospective study included consecutive subjects referred to the Department of Periodontology, affiliated with Timone Hospital of Aix‐Marseille University School of Dental Medicine (Pôle PROMOD‐ODONTO, Timone, AP‐HM, Marseille, France), between February 2023 and October 2024, for the treatment of one or more GR. All patients sought consultation for DH which led to an avoidance reaction and/or pain evaluated as a Schiff score of 2 or 3 [4].

All dentists assigned to data collection (including clinical assessments and computerized image analyses) were calibrated and trained prior to the start of the study. Patient data were recorded in the comprehensive medical management system of the hospital.

Ethical approval was granted by the Ethics Committee of XXX and the Protected Data Access Portal of Aix‐Marseille University (N° 2024‐12‐12‐05) and the Protected Data Access Portal of the Public Assistance Hospitals of Marseille (PADS25‐99), in accordance with the Declaration of Helsinki [20].

Patients who fulfilled the following inclusion criteria were selected: (1) patients aged 18 years or older who underwent at least one RC surgery; (2) initial diagnosis of gingival health on a reduced periodontium, with or without a history of periodontitis; (3) presence of at least one GR (RT1 or RT2) associated with DH which led to an avoidance reaction and/or pain (Schiff score 2 or 3); (4) DH refractory to desensitizing agents. The patient exclusion criteria were as follows: (1) unavailable patient data; (3) smoking patients; (4) pregnant or breastfeeding women.

### 2.2 Surgical Procedure

All procedures were performed by three trainee practitioners enrolled in the post‐graduate program for periodontal plastic surgery at Aix‐Marseille University: Dr. C.F., Dr. A.F., and Dr. R.O. Before surgery, all patients underwent a behavioral modification phase [21], which included oral and periodontal hygiene education. This phase involved demonstrations of efficient and atraumatic brushing techniques, as well as the use of calibrated interdental brushes and toothbrush heads with ultra‐soft bristles.

**FIGURE 1:**
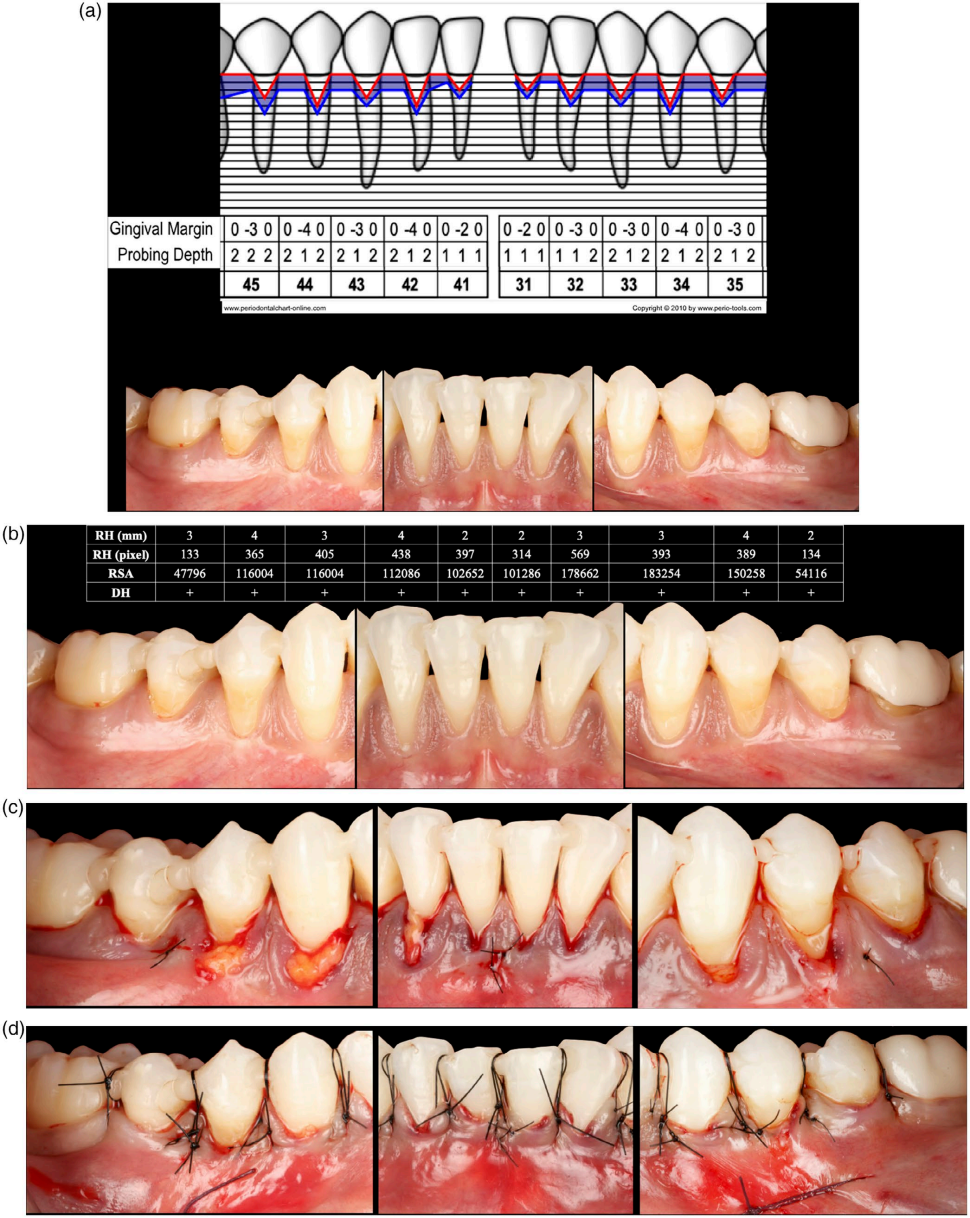
(A) Clinical situation of a 42‐year‐old patient, referred by her orthodontist for periodontal evaluation. The patient presented with healthy gingiva on a reduced periodontium and a history of periodontitis, as well as malpositioned and crowded anterior teeth. Full periodontal charting of the affected area was performed. Clinical criteria relevant to periodontal plastic surgery are summarized in Table 1. (B) Clinical situation showing heterogeneous RT2 recessions, with soft tissue thickness < 1 mm. Recession height (RH) in mm, recession height (RH) in pixels, recession surface area (RSA), and dentin hypersensitivity (DH) are reported in the corresponding table. (C) A modified tunnel technique [22] was performed with a connective tissue graft sutured (Ethilon 6/0) from tooth 45 to 35. (D) The previously created tunnel was coronally advanced and stabilized using double‐crossed sutures and suspended sutures (Ethilon 5/0).

Coronally advanced flaps or coronally advanced tunnel [22] associated with a connective tissue graft were performed for RC [23] (Figure 1C,D).

**TABLE 1:** Clinical criteria assessment preoperatively for periodontal plastic surgery for the patient in Figure 1A–D.

Tooth	Face		PPD (mm)			RH (mm)	Recession type	KTH (mm)	KTT (mm)	SCASS
	B	L	M	Mi	D					
45	x		2	2	2	3	RT2	3	> 1	2
44	x		2	1	2	4	RT2	0	> 1	2
43	x		2	1	2	3	RT2	1	< 1	2
42	x		2	1	2	4	RT2	1	< 1	2
41	x		1	1	1	2	RT2	3	< 1	2
31	x		1	1	1	2	RT2	3	< 1	2
32	x		1	1	2	3	RT2	2	< 1	2
33	x		2	1	2	3	RT2	1	< 1	2
34	x		2	1	2	4	RT2	1	< 1	2
35	x		2	1	2	3	RT2	2	> 1	2

Abbreviations: KTH, keratinized tissue height; KTT, keratinized tissue thickness; PPD, periodontal probing depth; RH, recession height; SCASS, Schiff Cold Air Sensitivity Scale.

### 2.3 Data Collection

#### 2.3.1 Measurement of DH

On the day of surgery and 6 months post‐operatively, DH was assessed according to the Schiff Cold Air Sensitivity Scale (SCASS) established by Schiff et al. [4].

A clinical evaporative air stimulus was applied to the dried tooth for one second from 1 cm.

Based on the patient's response, a score was assigned:

- *Score 0*: The patient does not react to the air stimulus.
- *Score 1*: The patient reacts to the air stimulus but does not request its cessation.
- *Score 2*: The patient reacts to the air stimulus and asks for it to be interrupted or moves away from it.
- *Score 3*: The patient reacts to the air stimulus, finds it painful, and requests its immediate cessation.

A score of 2 or 3 was recorded as the presence of significant DH, while a score of < 2 was considered as the absence of significant DH. This describes the dichotomous variable: presence/absence.

#### 2.3.2 Calculation of the RSA and Recession Height in Pixels Using ImageJ Software

**FIGURE 2:**
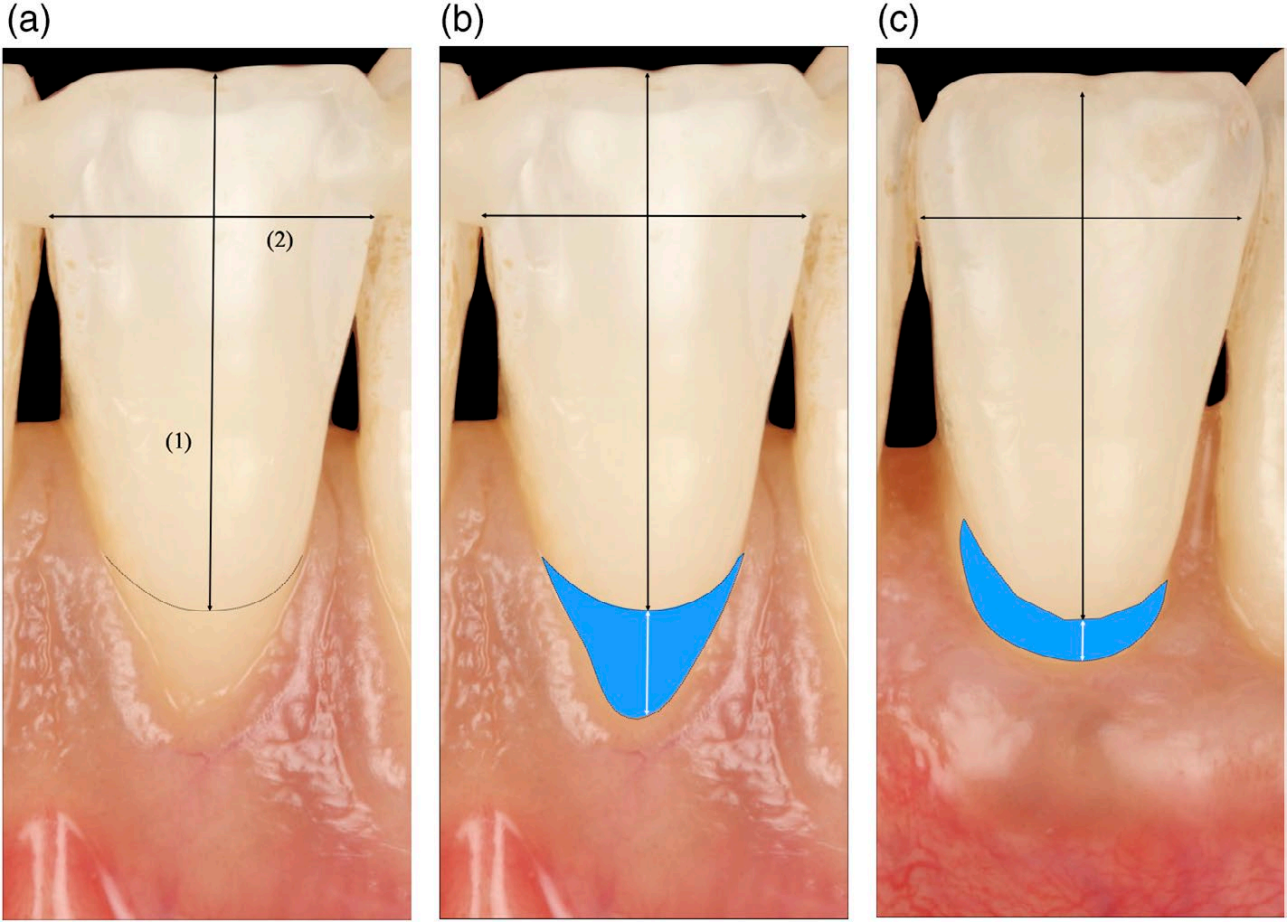
Measurements with ImageJ *software*. (A) Vertical (1) and horizontal (2) lines are drawn to check the reproducibility of the pictures. (B, C) The recession surface area (RSA) (blue surface) allowed for evaluating recession defects before and after surgery.

Each included tooth digital photograph was cropped, and the following two lines were drawn on a graphic tablet: (1) a straight apico‐coronal vertical line from the most apical point of the recession depth (RD) to the most coronal portion of the crown edge (Figure 2A); (2) a mesio‐distal horizontal line, at the widest part of the crown (Figure 2B). These two lines were used as references to check the reproducibility of the magnification. Then, the cemento‐enamel junction (CEJ) and the contour of the recession were drawn (Figure 2C) [19].

The RSA, measured in pixels, was used to calculate the root surface coverage percentage (RSCpix) using the following formula: ([(pre‐operative RSA − post‐operative RSA)/(pre‐operative RSA)] × 100).

Additionally, the recession height was delineated.

#### 2.3.3 Recession Height Measurement in Mm

A calibrated periodontal probe (PCPUNC15, Hu‐Friedy, Chicago, IL, USA) was used for precise clinical measurements of recession height under magnification glasses and a standardized lighting source to enhance accuracy. Recession height was measured clinically as the distance (in mm) from the CEJ to the most apical point of the gingival margin at the mid‐buccal aspect of the affected tooth. The PCPUNC15 probe was positioned parallel to the long axis of the tooth to minimize angulation errors. The measurement was recorded to the nearest millimeter mark on the probe.

### 2.4 Outcomes

In cases where the cemento‐enamel junction was not clearly visible clinically—particularly in teeth classified as Type B according to Pini‐Prato et al. [24]—the CEJ location was estimated during intraoral probing based on anatomical landmarks and gingival contour, and then projected onto the digital photographs for surface and height measurements.

The primary outcome of the study was the prevalence of DH (Schiff score ≥ 2) at 6 months post‐surgery. This classification allowed teeth to be grouped into those that no longer exhibited significant DH (DH−) and those that remained with significant DH (DH+).

The secondary outcomes included the comparison of prevalence and quantity (*Height* and *Surface*) of Root Coverage (RC) between the two DH groups:

1. *Prevalence of Complete Root Surface Coverage in pixels* (*CRSCpix*), defined as RSCpix = 100%.
2. *Prevalence of Complete Root Height Coverage in pixels* (*CRHCpix*) defined as *RHCpix* = *100%*.
3. *Prevalence of Complete Root Height Coverage in mm* (*CRHCmm*) defined as *RHCmm* = *100%*.
4. *Mean root surface coverage percentage* (*RSCpix%*), based on recession surface measured in pixels.
5. *Mean root height coverage percentage* (*RHCpix%*), based on recession height measured in pixels.
6. *Mean root height coverage percentage* (*RHCmm%*), based on recession height measured clinically in millimeters.

### 2.5 Statistical Analysis

At 6 months postoperatively, comparisons between teeth with (DH+) and without (DH−) significant DH were conducted for the three continuous variables: RSCpix, RHCpix, and RHCmm. Descriptive statistics for continuous variables: *RSCpix%*, *RHCpix%*, *RHCmm%* were reported as means ± standard deviations (mean ± SD). The distributions of RSCpix, RHCpix, and RHCmm were compared between the two groups using the Mann–Whitney *U* test. Categorical variables, including partial (0%–99.9%) and total (100%) RC, were analyzed using Fisher's exact test. Given the small sample size of the DH+ group (*n* = 5, 6.8%), non‐parametric tests were preferred over parametric alternatives to enhance the robustness of the results, and univariate logistic regression with Firth's correction was used to estimate odds ratios [25]. The significance level was set at *p* < 0.05. All statistical analyses were performed using SAS 9.4 (SAS Institute, Cary, NC).

**FIGURE 3:**
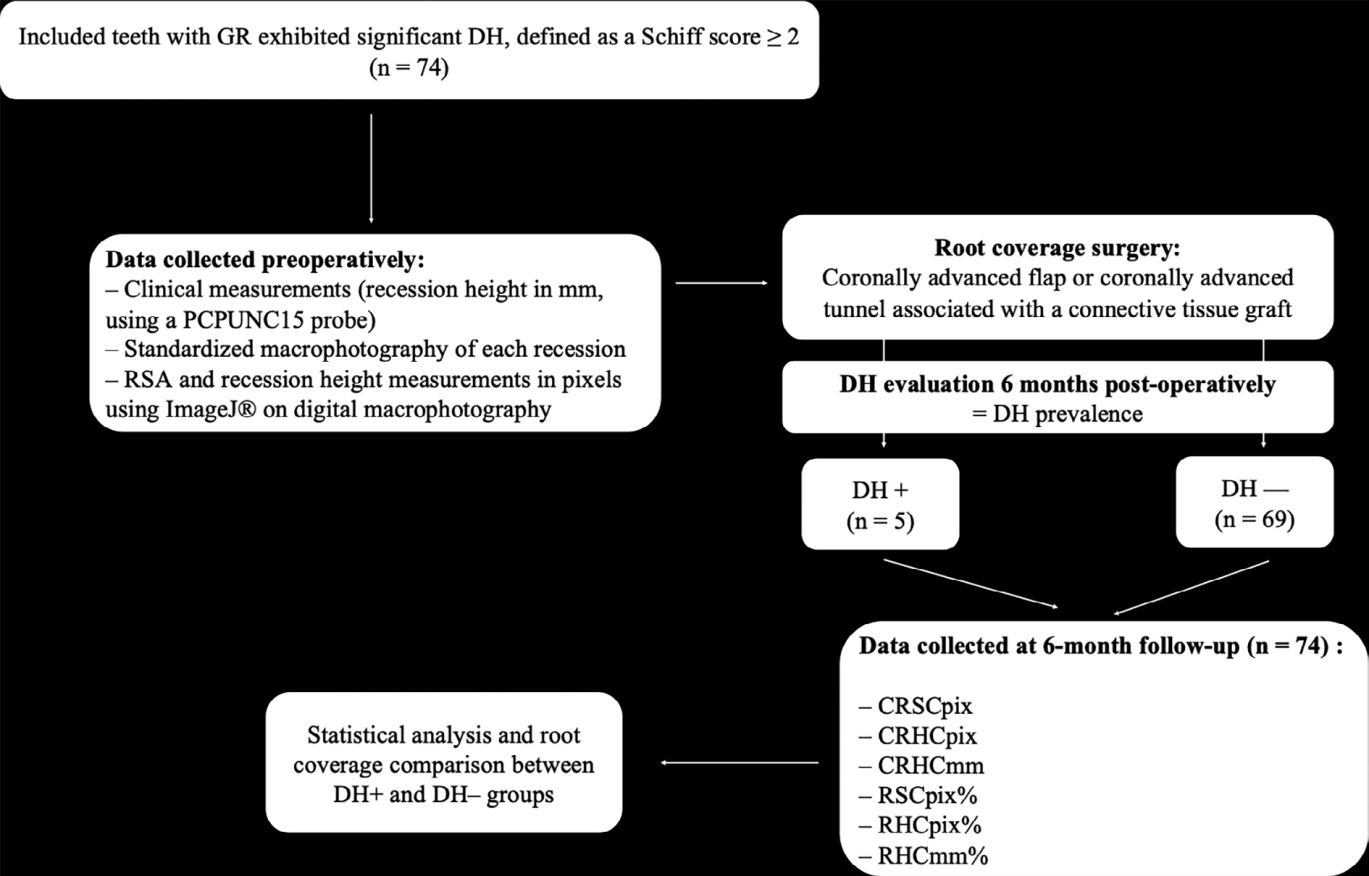
The flow chart of this study. CRHCmm, prevalence of complete root height coverage in mm defined as RHCmm = 100%; CRHCpix, prevalence of complete root height coverage in pixels defined as RHCpix = 100%; CRSCpix, prevalence of complete root surface coverage in pixels defined as RSCpix = 100%; DH, dentin hypersensitivity; GR, gingival recession; RHCmm, mean root height coverage percentage based on recession height measured clinically in millimeters; RHCpix%, mean root height coverage percentage based on recession height measured in pixels; RSCpix%, mean root surface coverage percentage based on recession surface measured in pixels.

A detailed flowchart (Figure 3) illustrates the inclusion/exclusion process and the timeline of assessments.

## 3 Results

### 3.1 Study Population

A total of 74 RT1 and RT2 were treated in the 14 included patients (49.4 ± 14.0 years) (Table 2).

**TABLE 2:** Patients and recessions characteristics [26].

	Patients (*n* = 14)	
	*n*	%
Age		
Mean (SD)	49.4 (14.0)	
Sex		
Women	9	64.3
Men	5	35.7

	Recessions (*n* = 74)	
	*n*	%
Type (Cairo)		
RT1	29	39.2
RT2	45	60.8

### 3.2 Outcomes

#### 3.2.1 RC and DH 6 Months After Periodontal RC Surgery

##### 3.2.1.1 Primary Outcomes

The prevalence of significant DH (DH+) was 6.8% (95% confidence interval [CI] [1.0%–12.5%]), while the absence of significant DH (DH−) was observed in 93.2% of treated teeth.

#### 3.2.2 Secondary Outcomes

1. The complete root surface coverage measured in pixels (CRSCpix) was obtained in 69.6% of DH−, significantly higher than in DH+ (0.0%, *p* = 0.0041).
2. The complete root height coverage measured in pixels (CRHCpix) was obtained in 63.8% of DH−, not significantly higher than in DH+ (20.0%, *p* = 0.0737).
3. The complete root height coverage measured in millimeters (CRHCmm) was obtained in 76.8% of DH−, not significantly higher than in DH+ (40.0%, *p* = 0.1033).
4. The mean root surface coverage percentage based on recession surface measured in pixels (RSCpix) was 86.6% ± 21.1% for the whole sample, and 88.3% ± 19.7% in DH−, significantly higher than 62.6% ± 28.2% in DH+ (*p* = 0.0031).
5. The mean root coverage percentage based on recession height measured in pixels (RHCpix) was 82.2% ± 28.7% for the whole sample, and 83.2% ± 28.3% in DH−, not statistically significant different compared to 68.8% ± 35.1% in DH+ (*p* = 0.0573).
6. The mean root coverage percentage based on recession height measured clinically in millimeters (RHCmm) was 87.2% ± 25.2% for the whole sample, and 88.2% ± 24.7% in DH−, not statistically significant different compared to 73.3% ± 30.8% in DH+ (*p* = 0.0503) (Table 3).

**TABLE 3:** Comparison of root coverage outcomes between teeth with (DH+) and without (DH−) postoperative significant DH (score of 2 or 3 according to the Schiff Cold Air Sensitivity Scale [SCASS] [4]).

	Post‐operative DH						All	
	DH−		DH+					
	*n*	*%*	*n*	*%*	*p*‐value^a^	Odds ratio 95% confidence interval	*n*	*%*
Overall prevalence	69	93.2	5	6.8	−	[1.0%−12.5%]	74	100.0%
RSCpix *Mean* ± SD	88.3 ± 19.7		62.6 ± 28.2		0.0031*		86.6 ± 21.1	
*CRSCpix*	48	69.6	0	0.0	0.0041*	0.04 [0.00;0.78]	48	64.9%
RHCpix *Mean* ± SD	83.2 ± 28.3		68.8 ± 35.1		0.0573		82.2 ± 28.7	
*CRHCpix*	44	63.8	1	20.0	0.0737	0.19 [0.03;1.32]	45	60.8%
RHCmm *Mean* ± SD	88.2 ± 24.7		73.3 ± 30.8		0.0503		87.2 ± 25.2	
*CRHCmm*	53	76.8	2	40.0	0.1033	0.22 [0.04;1.26]	55	74.3%

Abbreviations: CRHCmm, complete root height coverage in millimeters; CRHCpix, complete root height coverage in pixels; CRSCpix, complete root surface coverage in pixels; DH, dentin hypersensitivity; DH+, group with DH; DH−, group without DH; RHCmm, root height coverage in millimeters; RHCpix, root height coverage in pixels; RSCpix, root surface coverage in pixels.

^a^ Mann–Whitney *U* test for continuous variables and Fisher's exact test for categorical variables.

* Indicates statistical significance.

**FIGURE 4:**
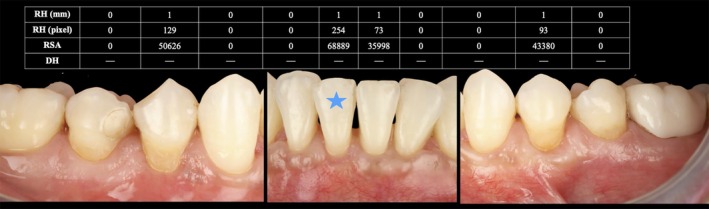
Clinical results obtained after 6 months, with a blue star symbol indicating the tooth‐level measurements shown in Figure 2. Recession height (RH) in mm, recession height (RH) in pixels, recession surface area (RSA), and dentin hypersensitivity (DH) are reported in the corresponding table.

The results are illustrated through the clinical case presented in Section 2 (Figure 4).

## 4 Discussion

In this retrospective study, periodontal plastic RC surgery resulted in the suppression of significant DH in 93.2% of cases after 6 months. In their meta‐analysis, Antezack et al. reported a 73.3% suppression rate using coronally advanced flaps combined with connective tissue grafts, underscoring the effectiveness of this approach not only for complete RC but also for DH suppression [17]. The superior results observed in our study may be attributed to the smaller sample size (74 vs. 1086).

The mean complete root surface coverage (CRSCpix) was 64.9% across the entire sample. Notably, there was no complete coverage in the DH+ group, supporting a causal relationship between exposed root surfaces and DH, as previously proposed by Clauser et al. [27]. Interestingly, only 69.6% of cases in the DH− group achieved complete root coverage, indicating that partial RC may also contribute to significant DH suppression. This aligns with findings from recent meta‐analyses and clinical trials suggesting that even partial root coverage can provide psychological or physiological benefits leading to DH suppression, as indicated by Evers et al. [28].

The complete root height coverage in pixels (CRHCpix) was 60.8%, and the corresponding height coverage in millimeters (CRHCmm) was 74.3%. These values are slightly higher than the 57.46% height coverage reported in the meta‐analysis by Tavelli et al. [29], likely due to variations in inclusion criteria, surgical techniques, and sample characteristics.

The discrepancy between CRHCpix and CRHCmm reflects a key clinical issue: traditional clinical assessment of gingival recession, typically based on intraoral measurement of the distance between the CEJ and the gingival margin using a periodontal probe (manual probing), tends to oversimplify the three‐dimensional nature of the defect and is prone to variability in probing pressure and anatomical interpretation. This can result in overestimated clinical success rates [30, 31, 32].

By contrast, ImageJ software allows for a more precise quantification of root surface coverage, particularly when small interproximal root areas remain exposed. This tool provides a reproducible and objective alternative to manual probing.

The mean root surface coverage based on surface area (RSCpix) was 86.6 ± 21.1 for the full sample, and 88.3 ± 19.7 in the DH− group—significantly higher than the 62.6 ± 28.2 observed in the DH+ group (*p* = 0.0031). Similarly, mean root height coverage in pixels (RHCpix) was 82.2 ± 28.7 overall, with 83.2 ± 28.3 in the DH− group and 68.8 ± 35.1 in the DH+ group, although this difference was not statistically significant (*p* = 0.0573).

Our mean RHCmm of 87.2 ± 25.2 was consistent with the results of Barootchi and Tavelli [33], who reported a mean coverage of 87.9 in height. No statistically significant difference in recession height coverage (in pixels or millimeters) was observed between DH+ and DH− groups, although the DH+ group consistently showed lower mean values. However, the significant difference observed in root surface coverage (RSCpix) suggests that surface area is a more sensitive indicator of clinical outcomes in cases involving DH [32].

Therefore, recession surface measurement offers a more discriminating method than linear height alone when evaluating gingival recession associated with DH. ImageJ provides a valid and accurate method for quantifying both parameters from digital photographs, improving over manual probing in assessing surgical success [18]. These advanced methods may enhance our understanding of DH suppression mechanisms, especially by emphasizing surface reduction over linear gingival height gain. Intraoral scanners, coupled with three‐dimensional (3D) analysis software, could allow clinicians to evaluate gingival margin shifts and recession defects with submillimetric precision and improved reproducibility [32].

The relationship between root surface coverage and DH suppression may also be influenced by other biological mechanisms, such as gingival phenotype thickening, pulpal defenses including calcification, secondary dentin formation, and sclerosis [34]. Furthermore, addressing etiological factors—such as promoting less traumatic and more effective brushing techniques and resolving inflammation—can also help mitigate DH [35].

A limitation of this study, which also reflects a common clinical challenge, is the difficulty in precisely identifying the CEJ when it is not clearly visible, as in Type B defects [24]. This uncertainty can compromise the accuracy of both recession height and surface measurements before and after root coverage procedures.

All participants (*n* = 14) presented with clinically significant DH that prompted consultation. DH was assessed on a tooth‐by‐tooth basis, and its suppression in 93.2% of cases suggests a tangible improvement in patients' quality of life, as supported by previous findings [7, 36, 37].

Given our focus on tooth‐specific outcomes, we opted for the validated Schiff score as a reliable measure of DH, which, although limiting direct comparison with studies using broader quality‐of‐life indices like the DHEQ [38] or OHIP [8], allowed for a more targeted clinical assessment.

## 5 Conclusion

Within the limitations of the present study, it was concluded that: the extent of root surface coverage achieved by surgical root coverage procedures played a key role in DH suppression; the quantification of the covered root surface area in pixels was more strongly associated with DH suppression than the measurement of recession height.

## Conflicts of Interest

The authors declare no conflicts of interest.

## Data Availability

The data that support the findings of this study are available from the corresponding author upon reasonable request.

## References

[1] H. J. Shiau , “Dentin Hypersensitivity,” Journal of Evidence‐Based Dental Practice 12, no. 3 Suppl (September 2012): 220–228. 10.1016/S1532-3382(12)70043-X 23040350

[2] G. R. Holland , M. N. Narhi , M. Addy , L. Gangarosa , and R. Orchardson , “Guidelines for the Design and Conduct of Clinical Trials on Dentine Hypersensitivity,” Journal of Clinical Periodontology 24, no. 11 (November 1997): 808–813. 9402502 10.1111/j.1600-051x.1997.tb01194.x

[3] K. Que , B. Guo , Z. Jia , Z. Chen , J. Yang , and P. Gao , “A Cross‐Sectional Study: Non‐Carious Cervical Lesions, Cervical Dentine Hypersensitivity and Related Risk Factors,” Journal of Oral Rehabilitation 40, no. 1 (2013): 24–32. 22882712 10.1111/j.1365-2842.2012.02342.x

[4] T. Schiff , E. Delgado , Y. P. Zhang , W. DeVizio , D. Cummins , and L. R. Mateo , “The Clinical Effect of a Single Direct Topical Application of a Dentifrice Containing 8.0% Arginine, Calcium Carbonate, and 1450 Ppm Fluoride on Dentin Hypersensitivity: The Use of a Cotton Swab Applicator Versus the Use of a Fingertip,” Journal of Clinical Dentistry 20, no. 4 (2009): 131–136. 19831166

[5] N. West , M. Davies , A. Sculean , et al., “Prevalence of Dentine Hypersensitivity, Erosive Tooth Wear, Gingival Recession and Periodontal Health in Seven European Countries,” Journal of Dentistry 150 (September 2024): 105364. 39317300 10.1016/j.jdent.2024.105364

[6] V. Goh , E. F. Corbet , and W. K. Leung , “Impact of Dentine Hypersensitivity on Oral Health‐Related Quality of Life in Individuals Receiving Supportive Periodontal Care,” Journal of Clinical Periodontology 43, no. 7 (July 2016): 595–602. 27028655 10.1111/jcpe.12552

[7] D. W. Douglas‐de‐Oliveira , G. P. Vitor , J. O. Silveira , C. C. Martins , F. O. Costa , and L. O. M. Cota , “Effect of Dentin Hypersensitivity Treatment on Oral Health Related Quality of Life—A Systematic Review and Meta‐Analysis,” Journal of Dentistry 71 (April 2018): 1–8. 29262305 10.1016/j.jdent.2017.12.007

[8] K. Bekes , M. T. John , H. G. Schaller , and C. Hirsch , “Oral Health‐Related Quality of Life in Patients Seeking Care for Dentin Hypersensitivity,” Journal of Oral Rehabilitation 36, no. 1 (2009): 45–51. 19207369 10.1111/j.1365-2842.2008.01901.x

[9] J. H. Bae , Y. K. Kim , and S. K. Myung , “Desensitizing Toothpaste Versus Placebo for Dentin Hypersensitivity: A Systematic Review and Meta‐Analysis,” Journal of Clinical Periodontology 42, no. 2 (February 2015): 131–141. 25483802 10.1111/jcpe.12347

[10] M. V. García Olazabal , L. E. P. Moya , R. W. C. Cirisola , et al., “Effect of Photobiomodulation on Dentin Hypersensitivity: A Randomized Controlled Double‐Blind Clinical Trial,” Clinical Oral Investigations 29, no. 1 (January 2025): 84. 39853488 10.1007/s00784-025-06149-z

[11] X. X. Liu , H. C. Tenenbaum , R. S. Wilder , R. Quock , E. R. Hewlett , and Y. F. Ren , “Pathogenesis, Diagnosis and Management of Dentin Hypersensitivity: An Evidence‐Based Overview for Dental Practitioners,” BMC Oral Health 20, no. 1 (August 2020): 220. 32762733 10.1186/s12903-020-01199-z PMC7409672

[12] N. X. West , A. Lussi , J. Seong , and E. Hellwig , “Dentin Hypersensitivity: Pain Mechanisms and Aetiology of Exposed Cervical Dentin,” Clinical Oral Investigations 17, no. Suppl 1 (March 2013): S9–S19.23224116 10.1007/s00784-012-0887-x

[13] P. Cortellini and N. F. Bissada , “Mucogingival Conditions in the Natural Dentition: Narrative Review, Case Definitions, and Diagnostic Considerations,” Journal of Clinical Periodontology 45, no. Suppl 20 (June 2018): S190–S198. 29926504 10.1111/jcpe.12948

[14] B. S. K. Bin Bahar , S. R. Alkhalidy , E. G. Kaklamanos , and A. E. Athanasiou , “Do Orthodontic Patients Develop More Gingival Recession in Anterior Teeth Compared to Untreated Individuals? A Systematic Review of Controlled Studies,” International Orthodontics 18, no. 1 (March 2020): 1–9. 31685434 10.1016/j.ortho.2019.08.025

[15] P. A. Heasman , R. Holliday , A. Bryant , and P. M. Preshaw , “Evidence for the Occurrence of Gingival Recession and Non‐Carious Cervical Lesions as a Consequence of Traumatic Toothbrushing,” Journal of Clinical Periodontology 42, no. Suppl 16 (April 2015): S237–S255. 25495508 10.1111/jcpe.12330

[16] D. M. Kim and R. Neiva , “Periodontal Soft Tissue Non‐Root Coverage Procedures: A Systematic Review From the AAP Regeneration Workshop,” Journal of Periodontology 86, no. 2 Suppl (February 2015): S56–S72. 25644300 10.1902/jop.2015.130684

[17] A. Antezack , R. Ohanessian , C. Sadowski , et al., “Effectiveness of Surgical Root Coverage on Dentin Hypersensitivity: A Systematic Review and Meta‐Analysis,” Journal of Clinical Periodontology 49, no. 8 (August 2022): 840–851. 35634650 10.1111/jcpe.13664

[18] S. Kerner , D. Etienne , J. Malet , F. Mora , V. Monnet‐Corti , and P. Bouchard , “Root Coverage Assessment: Validity and Reproducibility of an Image Analysis System,” Journal of Clinical Periodontology 34, no. 11 (November 2007): 969–976. 17877749 10.1111/j.1600-051X.2007.01137.x

[19] G. J. Kerner , S. Le Roch , D. Damman , and P. Bouchard , “Image Distortion of Intra‐Oral Photographs: The Root Coverage Model,” Journal of Clinical Periodontology 47, no. 7 (July 2020): 875–882. 32368811 10.1111/jcpe.13294

[20] K. Morris , “Revising the Declaration of Helsinki,” Lancet 381, no. 9881 (June 2013): 1889–1890. 23734387 10.1016/s0140-6736(13)60951-4

[21] M. C. Carra , L. Detzen , J. Kitzmann , J. P. Woelber , C. A. Ramseier , and P. Bouchard , “Promoting Behavioural Changes to Improve Oral Hygiene in Patients With Periodontal Diseases: A Systematic Review,” Journal of Clinical Periodontology 47, no. Suppl 22 (July 2020): 72–89. 10.1111/jcpe.13234 31912530

[22] S. Aroca , B. Molnár , P. Windisch , et al., “Treatment of Multiple Adjacent Miller Class I and II Gingival Recessions With a Modified Coronally Advanced Tunnel (MCAT) Technique and a Collagen Matrix or Palatal Connective Tissue Graft: A Randomized, Controlled Clinical Trial,” Journal of Clinical Periodontology 40, no. 7 (July 2013): 713–720. 23627374 10.1111/jcpe.12112

[23] M. Sanz , M. Simion , and Working Group 3 of the European Workshop on Periodontology , “Surgical Techniques on Periodontal Plastic Surgery and Soft Tissue Regeneration: Consensus Report of Group 3 of the 10th European Workshop on Periodontology,” Journal of Clinical Periodontology 41, no. Suppl 15 (April 2014): S92–S97. 24641004 10.1111/jcpe.12215

[24] G. Pini‐Prato , D. Franceschi , F. Cairo , M. Nieri , and R. Rotundo , “Classification of Dental Surface Defects in Areas of Gingival Recession,” Journal of Periodontology 81, no. 6 (June 2010): 885–890. 20450362 10.1902/jop.2010.090631

[25] D. Firth , “Bias Reduction of Maximum Likelihood Estimates,” Biometrika 80, no. 1 (1993): 27–38.

[26] F. Cairo , M. Nieri , S. Cincinelli , J. Mervelt , and U. Pagliaro , “The Interproximal Clinical Attachment Level to Classify Gingival Recessions and Predict Root Coverage Outcomes: An Explorative and Reliability Study,” Journal of Clinical Periodontology 38, no. 7 (July 2011): 661–666. 21507033 10.1111/j.1600-051X.2011.01732.x

[27] C. Clauser , M. Nieri , D. Franceschi , U. Pagliaro , and G. Pini‐Prato , “Evidence‐Based Mucogingival Therapy. Part 2: Ordinary and Individual Patient Data Meta‐Analyses of Surgical Treatment of Recession Using Complete Root Coverage as the Outcome Variable,” Journal of Periodontology 74, no. 5 (May 2003): 741–756. 12816306 10.1902/jop.2003.74.5.741

[28] A. W. M. Evers , L. Colloca , C. Blease , et al., “Implications of Placebo and Nocebo Effects for Clinical Practice: Expert Consensus,” Psychotherapy and Psychosomatics 87, no. 4 (2018): 204–210. 29895014 10.1159/000490354 PMC6191882

[29] L. Tavelli , S. Barootchi , T. V. N. Nguyen , M. Tattan , A. Ravidà , and H. L. Wang , “Efficacy of Tunnel Technique in the Treatment of Localized and Multiple Gingival Recessions: A Systematic Review and Meta‐Analysis,” Journal of Periodontology 89, no. 9 (2018): 1075–1090. 29761502 10.1002/JPER.18-0066

[30] M. Kuralt , R. Gašperšič , and A. Fidler , “Methods and Parameters for Digital Evaluation of Gingival Recession: A Critical Review,” Journal of Dentistry 118 (March 2022): 103793. 34481931 10.1016/j.jdent.2021.103793

[31] D. Schneider , A. Ender , T. Truninger , et al., “Comparison Between Clinical and Digital Soft Tissue Measurements,” Journal of Esthetic and Restorative Dentistry 26, no. 3 (2014): 191–199. 24341747 10.1111/jerd.12084

[32] N. Gkantidis , K. Dritsas , M. Ghamri , D. Halazonetis , and A. Sculean , “Methods for 3D Evaluation and Quantification of Gingival Recessions and Gingival Margin Changes: Advancements From Conventional Techniques,” Periodontology 2000 (November 2024). 10.1111/prd.12615 39552108

[33] S. Barootchi and L. Tavelli , “Tunneled Coronally Advanced Flap for the Treatment of Isolated Gingival Recessions With Deficient Papilla,” International Journal of Esthetic Dentistry 17, no. 1 (February 2022): 14–26. 35175005

[34] S. J. West and M. Davies , “Dentine Hypersensitivity,” Monographs in Oral Science 25 (2014): 108–122. 24993261 10.1159/000360749

[35] M. S. Tonetti , S. Jepsen , and Working Group 2 of the European Workshop on Periodontology , “Clinical Efficacy of Periodontal Plastic Surgery Procedures: Consensus Report of Group 2 of the 10th European Workshop on Periodontology,” Journal of Clinical Periodontology 41, no. Suppl 15 (April 2014): S36–S43. 24640999 10.1111/jcpe.12219

[36] É. B. S. de Carvalho , R. Ferreira , B. O. Azuaga , et al., “Impact of Subepithelial Connective Tissue for Root Coverage on Brazilian Patients' Quality of Life: A Longitudinal Clinical Study,” Journal of the International Academy of Periodontology 23, no. 2 (April 2021): 99–105. 33929810

[37] F. Graziani and G. Tsakos , “Patient‐Based Outcomes and Quality of Life,” Periodontology 2000 83, no. 1 (June 2020): 277–294. 32385874 10.1111/prd.12305

[38] S. R. Baker , B. J. Gibson , F. Sufi , A. Barlow , and P. G. Robinson , “The Dentine Hypersensitivity Experience Questionnaire: A Longitudinal Validation Study,” Journal of Clinical Periodontology 41, no. 1 (2014): 52–59. 24117696 10.1111/jcpe.12181

